# Clinical effect of psychological nursing and nutritional intervention in radiopharmaceutical therapy of patients with thyroid carcinoma

**DOI:** 10.12669/pjms.40.9.9020

**Published:** 2024-10

**Authors:** Jing Chen, Yuanzheng Liu

**Affiliations:** 1 Jing Chen, Department of Nuclear Medicine, Baoding No.1 Central Hospital, Baoding, Hebei, 071000, China; 2 Yuanzheng Liu, Western Medicine Pharmacy Department, Baoding No.1 Central Hospital, Baoding, Hebei, 071000, China

**Keywords:** Psychological nursing, Nutritional intervention, Thyroid carcinoma, Radiotherapy, Nursing efficacy

## Abstract

**Objective:**

To investigate the clinical value of psychological nursing and nutritional intervention in radiopharmaceutical therapy of patients with thyroid carcinoma.

**Methods:**

This was retrospective study. One hundred and twenty patients with thyroid carcinoma were included at Baoding No.1 Central Hospital from May 10, 2021 to July 10, 2023 to as subjects and randomly divided into the control group and the experimental group (n=60 each group). Patients in the control group were given conventional nursing, while those in the experimental group received psychological nursing and nutritional intervention. The differences in quality of life, compliance, satisfaction and cognitive level between the two groups before and after intervention were compared.

**Results:**

After intervention, the scores of emotional functions, role function, cognitive function, physical function and social function of the experimental group were significantly higher than those of the control group, with statistically significant differences (P= 0.00); The compliance of the experimental group was 100%, which was significantly higher than 90% of the control group, with a statistically significant difference (P= 0.01). Moreover, the scores of SAS and SDS in the experimental group were significantly lower than those in the control group after intervention, with a statistically significant difference (P= 0.00).

**Conclusion:**

Psychological nursing and nutritional intervention may result in a variety of benefits for patients with thyroid carcinoma receiving radiopharmaceutical therapy, such as effectively ameliorating the quality of life of patients, improving the cognitive level of patients with the disease and treatment compliance, reducing anxiety and depression, and enhancing patient satisfaction with nursing.

## INTRODUCTION

Thyroid carcinoma, a clinically common thyroid malignancy, has been on the rise globally in terms of incidence over the past decade. However, its clinical efficacy is markedly superior to other cancers, especially the five years survival rate of patients receiving radioactive therapy, whose five years survival rate can be as high as over 90%.^1^ Currently, thyroidectomy + radioiodine therapy + levothyroxine replacement therapy is regarded as the standard method for the treatment of thyroid carcinoma.^2^ Patients with thyroid carcinoma usually show varying degrees of physical and psychological discomfort, such as low mood, depression, anxiety, and even fear, posing a negative threat to rehabilitation.^3^ In addition, malnutrition and weight loss are common phenomena in those with malignant tumors.^4^ Studies have revealed an incidence of malnutrition as high as 40% to 80% in those with malignant tumors during therapy, with more than 50% of those suffering obvious weight loss, which is significantly associated with patient death.^5^ It has been confirmed in numerous studies that those with severe weight loss are often associated with poor quality of life, treatment tolerance, and prognosis.^6^

Recent years have witnessed the continuous development of nursing science, and the conventional nursing model has been unable to cope with the radiopharmaceutical therapy of patients with thyroid carcinoma. Sound nursing as well as psychological and nutritional intervention may improve the psychological and nutritional status of patients, and improve their quality of life during therapy. In this study, 120 patients with thyroid carcinoma who received radiopharmaceutical therapy were given psychological care and nutritional intervention, investigated the clinical value of psychological nursing and nutritional intervention in radiopharmaceutical therapy of patients with thyroid carcinoma.

## METHODS

This was retrospective study. One hundred and twenty patients with thyroid carcinoma were included at Baoding No.1 Central Hospital from May 10, 2021 to July 10, 2023 and randomly divided into the control group and the experimental group, with 60 cases in each group. Patients’ data were collected from the electronic medical record system of our hospital, including baseline characteristics, medical history, physical examination and laboratory examination. Patients in the experimental group were given psychological nursing and nutritional intervention during the perioperative period, while those in the control group were given conventional specialist nursing intervention during the same period. No statistically significant differences were observed between the two groups in terms of general data, which were comparable (Table-I).

**Table-I:** Comparative analysis of general data between the experimental group and the control group(*χ̅*±*S*) n=60.

Indicator	Experimental group	Control group	t/χ^2^	P
Age (years old)	65.37±7.21	65.85±7.43	0.40	0.72
Female (cases, %)	36 (60%)	38 (63%)	0.14	0.71
Duration of disease (years)	3.43±0.63	3.52±0.32	0.78	0.33
BMI (kg/m^2^)	22.08±1.25	21.84±1.08	1.13	0.26
***Pathological type***				
Papillary carcinoma (cases, %)	37 (62%)	35 (58%)	0.13	0.67
Follicular carcinoma (cases, %)	13 (22%)	16 (27%)	0.41	0.52
Medullary carcinoma (cases, %)	10 (16%)	9 (15%)	0.08	0.80
***Education level***				
Junior high school and below (cases, %)	28 (47%)	25 (42%)	0.30	0.58
High school (cases, %)	19 (32%)	20 (33%)	0.04	0.85
College or above (cases, %)	13 (21%)	15 (25%)	0.19	0.67

P > 0.05.

### Ethical Approval:

The study was approved by the Institutional Ethics Committee of Baoding No.1 Central Hospital (No: [2022]060; Date: November 03, 2022), and written informed consent was obtained from all participants.

### Inclusion criteria:

- Patients diagnosed with thyroid carcinoma by pathology;
- Those with indications for radiotherapy^7^;
- Those aged 45 to 75;
- Those who were conscious, had good treatment compliance, and could actively cooperate with treatment and the implementation of this study;
- Those who gave informed consent and voluntarily participated in this study;
- Those with complete clinical data.

### Exclusion criteria:

- Patients with severe diseases of other organs and systems;
- Those with severe liver and kidney function injury or other malignant tumors;
- Those with coagulation dysfunction;
- Pregnant and lactating women;
- Those who were cognitively unclear and unable to effectively and reasonably express their own wishes and who were unable to cooperate with this study;
- Those with severe mental disorders or cognitive impairment;
- Patients with coronary heart disease, diabetes and other basic diseases;
- Those with poor treatment compliance who could not cooperate with this study.

Patients in the control group were given conventional nursing. Prior to treatment, visits were conducted by physicians to patients, and pre-treatment examinations were completed to assess the patients’ physical condition and guide them to prepare for treatment. After treatment, the changes in condition and vital signs were monitored, medication was guided according to medical orders, and patients were instructed to take more rest. Patients were informed of the implications of treatment. During hospitalization, the patients were instructed to use medications rationally, and informed of daily hygiene knowledge such as drinking more water, eating acidic food, defecation and urination. Before the patients were discharged from the hospital, they were taught the knowledge of disease care after discharge, and were guided in the correct use of medication and radiation protection.

Patients in the experimental group were given psychological nursing and nutritional intervention. The details are as follows:

### Health education:

Psychological counseling was given to the patients according to their condition, age and other background information. Besides, the preoperative and postoperative nursing contents were explained to the patients to ensure that they could achieve standard self-management.

### Psychological intervention:

Visual media was installed in the isolation ward to facilitate patients to contact their families through video calls. Nurses relieved the patients’ tension and anxiety by one-to-one conversation to relieve their loneliness. Psychological intervention was given 3-5 times a week, 30 min each time.

Patients were encouraged to drink water and excreta properly within one week after treatment to avoid radiation to internal organs. In addition, the utensils that the patients had touched were washed repeatedly, and they were transferred to the protective ward to isolate them from the surrounding people.

### Nutritional intervention:

Patients took vitamin C sublingually or chewed acidic chewing gum to control the deterioration of the condition, and the daily drinking water was controlled to more than 2 L. They were instructed to eat more high-calorie, high-protein and other iodine-free diets, and were prohibited from drinking strong tea, coffee, etc.

### Discharge guidance:

Patients were instructed to avoid contact vulnerable groups such as infants and pregnant women within two weeks after discharge, and to review physiological indicators four to six weeks after discharge. Both groups were follow-up for six months.

### Observation indicators:

- Comparative analysis of quality of life after intervention: The SF-36 scale^8^ was used to evaluate and compare the quality of life of the two groups, including five dimensions of emotional function, role function, cognitive function, physical function and social function. The higher the score, the higher the quality of life.^9^
- Compliance was divided into three levels based on patients’ cooperation with physicians: compliance, basic compliance and non-compliance. Compliance = (compliance + basic compliance)/number of cases × 100%.
- Comparative analysis of anxiety and depression scores: The Self-rating anxiety scale (SAS) and the self-rating depression scale (SDS)^10^ were employed to evaluate the emotional changes of the two groups before and after intervention. The lower the score, the better the emotional state.
- The patient satisfaction questionnaire short form (PSQ-18)^11^ was utilized to conduct a comparative analysis of the patients’ satisfaction before and after intervention, including very satisfied, relatively satisfied, satisfied, uncertain and dissatisfied. Total satisfaction= (very satisfied + relatively satisfied + satisfied)/total number of cases × 100%.
- Comparative analysis of cognitive level before and after intervention: A self-made scale was used to assess the cognitive level, including basic knowledge of the disease, precautions after treatment, daily diet, and precautions during rehabilitation after discharge. The total score of the scale as set at 100, with higher scores indicating higher cognitive levels.

### Statistical analysis:

According to the data of each indicator in the pre-survey, the sample size is estimated by 95% confidence interval, and the largest one is the sample size of the study. The sample size required for each group was≥60 cases on the basis of Fisher exact probability. All data in this study were statistically analyzed using SPSS 20.0 software, and measurement data were expressed as(*χ̅*±*S*). Two independent sample *t-test* was utilized for inter-group data analysis, paired *t-test* or analysis of variance was employed for intra-group data analysis, and χ^2^ test was used for rate comparison. *P* < 0.05 was considered a statistically significant difference.

## RESULTS

After intervention, the scores of emotional functions, role function, cognitive function, physical function and social function of the experimental group were significantly higher than those of the control group, with statistically significant differences (*P*=0.00) (Table-II). The compliance of the experimental group was 100%, which was significantly higher than 90% of the control group, with a statistically significant difference (*P*= 0.01) (Table-III).

**Table-II:** Comparative analysis of quality of life scores between the two groups before and after intervention (*χ̅*±*S*) n=60.

Group	Emotional function	Cognitive function	Physical function	Role function	Social function
Experimental group	7.65±1.47	5.63±1.58	7.63±1.51	4.87±0.46	5.69±1.43
Control group	6.10±1.04	3.38±1.02	6.20±1.47	4.51±0.30	4.58±1.82
*t*	6.67	9.21	5.27	5.08	3.72
*p*	0.00	0.00	0.00	0.00	0.00

P > 0.05.

**Table-III:** Comparative analysis of the compliance of the two groups after intervention (*χ̅*±*S*) n=60.

Group	Compliance	Basic compliance	Non-compliance	Compliance (%)
Experimental group	55	5	0	60 (100%)
Control group	43	11	6	54 (90%)
*χ* ^2^				6.32
*P*				0.01

P > 0.05.

No statistically significant differences were observed in the SAS and SDS levels between the two groups before intervention(*P* > 0.05). After intervention, the scores of SAS and SDS in the experimental group were significantly lower than those in the control group, with a statistically significant difference (*P*= 0.00) (Table-IV). The satisfaction of the experimental group was 100%, which was significantly higher than 85% of the control group, with a statistically significant difference (*P*= 0.00) (Table-V).

**Table-IV:** Comparative analysis of emotional status between the two groups before and after intervention (*χ̅*±*S*) n=60.

Indicator		Experimental group*	Control group	t	p
SAS	Before intervention	57.45±5.22	56.86±5.37	0.61	0.54
	After intervention*	42.58±5.12	45.71±5.23	3.31	0.00
SDS	Before intervention	52.06±5.14	52.43±5.20	0.40	0.68
	After intervention*	43.27±5.48	45.93±5.31	3.75	0.00

* P < 0.05.

**Table-V:** Comparative analysis of patient satisfaction between the two groups (*χ̅*±*S*) n=60.

Group	Very satisfied	Relatively satisfied	Satisfied	Uncertain	Not satisfied	Total satisfaction*
Experimental group	48	7	5	0	0	60 (100%)
Control group	31	15	5	3	6	51 (85%)
*χ* ^2^						9.73
*P*						0.00

* P < 0.05.

No significant differences were observed between the two groups in terms of cognitive scores before intervention(*P*=0.87). After intervention, the cognitive level of both groups increased compared with that before intervention(*P*=0.00), and the degree of increase in the experimental group was significantly higher than that in the control group, with a statistically significant difference (*P*= 0.00) (Table-VI).

**Table-VI:** Comparative analysis of cognitive levels between the two groups before and after intervention (*χ̅*±*S*) n=60.

Group	Before intervention	After intervention*	t	p
Experimental group	43.57±10.48	57.36±10.83	7.15	0.00
Control group	43.80±10.31	51.18±10.30	3.83	0.00
*t*	0.12	3.20		
*P*	0.87	0.00		

* P < 0.05.

## DISCUSSION

It was confirmed in our study that the scores of emotional functions, role function, cognitive function, physical function and social function of the experimental group were significantly higher than those of the control group after intervention, with statistically significant differences (*P* = 0.00); The compliance of the experimental group was 100%, which was significantly higher than 90% of the control group, with a statistically significant difference (*P* = 0.01). Moreover, the scores of SAS and SDS in the experimental group were significantly lower than those in the control group after intervention, with a statistically significant difference (*P* = 0.00). There is also a large amount of research data pointing out that psychological nursing and nutritional intervention modes are more in line with the concept of humanized nursing. They are conducive to guiding patients to timely resolve negative emotions during nursing to ensure that they respond to the prognosis with a positive attitude. From the perspective of nursing work, patients can establish a good relationship with nurses based on psychological nursing and intervention, so as to better feel the care from medical staff. This raises the cooperation of patients to another level, which is crucial for the comprehensive development of nursing work and the adequate and reasonable maintenance of patient health.^12^ More studies by Liu et al.^13^ believe that reasonable psychological nursing can significantly improve patients’ prognostic quality of life score, further confirming the clinical application value of this nursing mode.

However, patients often experience the adverse symptoms of thyroid carcinoma and related surgical treatment before undergoing isolation treatment. This is also the main reason why they are burdened physically and psychologically and are prone to negative emotions.^14^ To make matters worse, iodine-131 therapy needs to be carried out in isolation, which prevents family members from visiting patients during treatment. As a result, patients have no reasonable way to vent their overstocked emotions, which is not conducive to the effective protection of their psychological state.^15^ Schrepf et al.^16^ argues that cancer patients are emotionally and cognitively impaired and therefore suffer from severe mental health problems. Studies have pointed out that cancer patients have a fear of disease recurrence.^17^

A sound nursing plan needs to be developed to improve the physical and psychological health of patients and to prevent treatment from affecting them.^18^ To reasonably respond to the above-mentioned related issues, nurses should pay close attention to the psychological state of patients in the nursing process. This can not only effectively improve patients’ ability to control their own emotions, but also provide assistance in relieving their negative mentality. Psychological nursing and nutritional intervention contribute to correcting patients’ wrong cognition via psychological intervention and nutrition, and help them increase their self-management knowledge. This approach facilitates clinical caregivers to help patients identify, assess, and modify inaccurate or otherwise unhelpful thoughts related to emotional distress.^19^ By doing so, patients can achieve the same thinking as the medical staff.

Tumor is a chronic wasting disease. Even at the lowest level of activity, patients still have a high metabolic rate and require more nutrients than normal people.^20^ In case of nutrient deficiency, the body’s cell and tissue repair capabilities decrease, resulting in low body tolerance. Studies have reported that reduced treatment tolerance in malnourished patients may lead to inadequate treatment, resulting in lower dose, treatment interruption or even discontinuation.^21^ Many other studies have also confirmed that malnutrition can cause decreased immune function, reduced anti-cancer ability, physical weakness, aggravation of cancer-related fatigue, increased discomfort, and decreased quality of life.^22^ In this study, patients with thyroid cancer were given nutritional intervention during treatment. The results showed that after intervention, the quality of life, satisfaction and cognitive level of patients in the experimental group were significantly better than those in the control group (*P*<0.05). The reason is that individualized nutrition of dietary energy supply combined with professional nutritional guidelines and patients’ own energy consumption for individualized nutrition and dietary management can significantly improve their nutritional status, enhance physical fitness, and improve immunity. While enhancing anti-tumor and anti-external damage ability, this combined therapy can improve radiotherapy tolerance and reduce the side effects of the tumor itself and treatment. All these indicate that nutritional intervention plays a crucial role in the comprehensive treatment of tumors.

### Limitations

Nonetheless, there are certain limitations in this study: fewer samples are recruited and follow-up is not included in the study. In future clinical practice, the sample size will continue to increase and the follow-up content will be further improved, in order to more objectively evaluate the advantages and disadvantages of the intervention program and benefit more patients.

## CONCLUSIONS

Psychological nursing and nutritional intervention may result in a variety of benefits for patients with thyroid carcinoma receiving radiopharmaceutical therapy, such as reducing patients’ negative emotions and improving their nutritional status. Furthermore, this combined therapy improves patients’ quality of life, cognitive level and compliance with treatment, may reduce anxiety and depression, and enhance patient satisfaction with nursing, which is of certain clinical application value.

### Authors’ Contributions:

**JC** carried out the studies, data collection, drafted the manuscript, are responsible and accountable for the accuracy or integrity of the work.

**YL** performed the statistical analysis , review and participated in its design.

All authors read and approved the final manuscript.
